# High-biologically effective dose palliative radiotherapy for a tumor thrombus might improve the long-term prognosis of hepatocellular carcinoma: a retrospective study

**DOI:** 10.1186/s13014-017-0831-y

**Published:** 2017-05-31

**Authors:** Xiang-quan Kong, Ya-ping Dong, Jun-xin Wu, Jun-yan He, Yu-yin Le, Kai-xin Du, Qing-qin Peng, Jin-luan Li

**Affiliations:** Department of Radiation Oncology, Fujian Medical University Cancer Hospital, Fujian Cancer Hospital, 420 Fuma Rd, Jinan District, Fuzhou, 350014 China

**Keywords:** Biologically effective dose, Hepatocellular carcinoma, Overall survival, Prognostic factor, Tumor thrombus

## Abstract

**Background:**

This study aimed to highlight the type of tumor thrombus and identify the prognostic factors influencing the long-term survival outcomes in patients with hepatocellular carcinoma (HCC) having a tumor thrombus. A tumor thrombus in HCC is associated with poor prognosis.

**Methods:**

Eighty patients diagnosed with HCC having a tumor thrombus between May 2006 and April 2014 were enrolled in this study. Age, gender, clinical characteristics, laboratory findings, Child-Pugh classification, performance status (ECOG), types of tumor thrombi, radiotherapy method, biologically effective dose (BED), and primary treatment method were analyzed to identify the prognostic factors associated with the overall survival (OS) rates. Statistical analyses were performed using SPSS version 19.0.

**Results:**

The median follow-up duration was 24 months (range 6–90). The 1-, 3-, and 5-year OS rates of the patients were 77.6%, 37.6%, and 18.8%, respectively. On univariate analysis, gender, radiotherapy method, BED, types of tumor thrombi, Child-Pugh classification, ECOG, and total bilirubin were associated with OS (*P* < 0.001, *P* = 0.001, *P* = 0.016, *P* = 0.003, *P* < 0.001, *P* < 0.001, *P* = 0.039, respectively). The prognostic factors for OS in multi-variate analyses were gender (*P* < 0.001), BED (*P* = 0.044), Child Pugh classification (*P* = 0.020), performance status (ECOG) (*P* = 0.004), and types of tumor thrombi (*P* = 0.001). The median OS for the high-BED group was better than that for the low-BED groups (42 months vs. 19 months, *P* = 0.016).

**Conclusions:**

Gender, BED, performance status (ECOG), Child-Pugh classification, and types of tumor thrombi seemed to affect OS, and a stepwise decrease in survival was observed with the types of tumor thrombi ranging from I to IV. High-BED palliative radiotherapy might improve the long-term outcomes for patients with HCC having a tumor thrombus.

## Background

Hepatocellular carcinoma (HCC) remains one of the most challenging tumors to treat and is a highly lethal cancer [1]. A tumor thrombus can be found in 10%–40% of locally advanced HCC during diagnosis, leading to poor prognosis [2]. Treatment strategies for HCC with a tumor thrombus are limited [3]. The treatment modality should be multidisciplinary and comprehensive, including radiotherapy (RT), transcatheter arterial chemoembolization (TACE), hepatic resection, and targeted therapy [4–7]. Although combined-modality approaches might have a prolonged survival, many patients with a tumor thrombus die relatively quickly and the standard treatment remains unknown. Surgical resection is controversial for patients with HCC having a tumor thrombus, which might lead to a residual tumor [8]. Some studies proposed that TACE might be a promising procedure in unresectable HCC with a tumor thrombus [9]. Whether TACE aggravates hepatic infarction or acute hepatic failure, especially in patients with a tumor thrombus, remains unanswered [10]. With the development of radiotherapy techniques, a few studies showed that external-beam radiation therapy (EBRT) might play a positive role in patients with a tumor thrombus [11]. Although RT might improve treatment outcomes, the optimal radiation dose remains uncertain. Till now, rare studies have been launched for a tumor thrombus of HCC. Also, no consensus on the type of tumor thrombus and its prognostic value has been achieved. The tumor thrombus system might be helpful for selecting the optimal treatment strategies [12].

This study attempted to assess the types of tumor thrombi and identify the prognostic factors influencing overall survival (OS) in HCC with a tumor thrombus.

## Methods

### Patients

This retrospective study analyzed 80 consecutive patients with HCC complicated with a tumor thrombus in Fujian Cancer Hospital from November 2006 to May 2016. The diagnosis of HCC was made using the American Association for the Study of Liver Disease guidelines [13]. A tumor thrombus in HCC was identified by magnetic resonance imaging (MRI), ultrasonography, and computed tomography (CT). Pretreatment assessment consisted of history and physical examination, liver function tests, blood tests, alpha-fetoprotein (AFP), and whole-body bone scan.

### Inclusion criteria

The inclusion criteria were: (1) patients with HCC complicated with a tumor thrombus; (2) patients were categorized as Eastern Cooperative Oncology Group (ECOG) status from 0 to 3; (3) Child-Pugh class A-B; (4) other severe systemic disease.

### Treatment

All 80 patients received TACE, of which 64 patients were treated using RT. Of these, 54 patients underwent accurate radiotherapy (A-RT), and the other 10 patients received two-dimensional radiotherapy (2D-RT). Intensity-modulated radiation therapy and three-dimensional conformal radiation therapy (3D-CRT) were defined as A-RT in this study. Also, 16 patients were treated with TACE alone in the non-RT group. TACE with the guidance of digital subtraction angiography was performed in all patients.

Ten patients were treated with 2D-RT. The design of 2D-RT was based on the CT scan of abdomen. The area of the liver radiation field was generally < 120 cm^2^. The dose of 2D-RT ranged from 30–50 Gy with 2–3 Gy per fraction. Fifty-four patients were treated with A-RT in our study. The dose of A-RT ranged from 40–60 Gy with 2–3 Gy per fraction. The patients were placed in the supine position, and the arms were kept above the head in the alpha cradle. The customizable thermoplastic positioning membrane and vacuum cushion could improve the positioning accuracy and strengthen position fixation in the radiotherapy. The image was transmitted to the 3D-CRT system. The gross tumor volume (GTV) consisted of the primary tumor and tumor thrombus, which were contoured on the planning CT images. The boundary of clinical target volume (CTV) was GTV plus 5–10 mm and the planning target volume (PTV) added a margin of 7 mm to the CTV to compensate for daily setup errors and target motion. The mean liver dose was kept less than 28 Gy, and no more than 50% of normal liver can receive ≥30 Gy (V30 ≤ 50%). If the patient had a concomitant disease of cirrhosis, the normal liver received a mean dose of <24 Gy.

Organs at risk (OARs): kidneys, mean dose to both kidneys must be <18 (V18 < 30%); spinal cord, maximum dose ≤40 Gy; duodenum maximum dose ≤45–50Gy (V45 ~ V50 < 10%); small intestine, maximum dose ≤45–50 Gy (V20 ~ V30 < 50%); stomach, maximum dose <50 Gy; no more than 25% of right lung receive ≥20Gy (V20 ≤ 25%).

A daily radiation dose of 2–3 Gy was administered using 6- or 15-MV x-rays from a linear accelerator. The biological effectiveness of radiation schedule could be compared with the biologically effective dose (BED) $$ \left(\begin{array}{c}\hfill BED= n\times d\left(1+\frac{d}{\raisebox{1ex}{$\alpha $}\!\left/ \!\raisebox{-1ex}{$\beta $}\right.}\right)-\frac{\alpha}{\gamma}\times \left( T- Tk\right)\hfill \\ {}\hfill \frac{\alpha}{\gamma}=\frac{0.6 Gy}{d}, T k=7 d, T=\mathrm{total}\ \mathrm{treatment}\ \mathrm{elapsed}\ \mathrm{days}\hfill \end{array}\right) $$. The BED ranged from 38.04 to 72.27 Gy, assuming an α/β ratio of 11.2 Gy for HCC [14], and the median of BED was 58.9 Gy.

Types I–III of tumor thrombi were categorized according to the Cheng’s classification: type I, a tumor thrombus involving the second branch of the portal vein; type II, a tumor thrombus involving the first branch; and type III, a tumor thrombus invading the trunk of portal vein [12]; and type IV, a tumor thrombus extending to the right atrium or inferior vena cava [15].

A total of 80 patients with a tumor thrombus were retrospectively evaluated.

### Follow-up

The data of patients were retrospectively analyzed. Two radiologists evaluated the radiotherapeutic response of tumor thrombus with CT or MRI according to the modified Response Evaluation Criteria in Solid Tumors (mRECIST) within 3 months after RT [16]. The response rate (RR) was defined as the sum of complete response (CR) and partial response (PR). Treatment related toxicity was evaluated with the Common Terminology Criteria for Adverse Events (CTCAE v4.0) [17]. Blood tests and liver function tests were assessed weekly during RT and then monthly. Survival was defined as the period from the diagnosis of tumor thrombus to death or the date of the last follow-up.

### Statistical analysis

The primary endpoint of this study was OS. Radiotherapeutic response was compared by the χ^2^-test. Groups were compared using the log-rank test. The Kaplan-Meier method was used to estimate the probability of survival. Also, the prognostic influences of tumor thrombus were compared using the log-rank test. All tests of *P* values were two sided, and *P* values less than 0.05 were statistically significant. The independent predictors for OS were calculated using the Cox proportional hazards model. All analyses were performed using SPSS version 19.0 (SPSS, IL, USA).

## Results

### Patient characteristics

Eighty cases were included for analysis in the study. The characteristics of patients and tumor are shown in Table 1. The median age of the patients was 54 years (range 30–72 years). Of these patients, 75 (97.3%) were males and 5 (6.3%) were females. Moreover, 54 patients (67.5%) received A-RT, 10 patients (12.5%) received 2D-RT, and 16 patients (20.0%) received TACE alone. The median of BED was 58.9 Gy. Of all the patients, 42 (52.5%) had a high BED level (≥58.9 Gy), 22 (27.5%) had a low BED level (<58.9 Gy) and 16 patients (20.0%) had 0 Gy. Again, 12 patients (15%) had type I, 28 patients (35%) had type II, 18 patients (22.5%) had type III, and 22 patients (27.5%) had type IV tumor thrombus. Considering performance status (PS) before RT, 59 (73.8%) of patients were ECOG 0–1, 21 (26.2%) were ECOG 2–3. Regarding Child-Pugh classification before RT, 50 (62.5%) of patients had class A, 30 (37.5%) had class B. There were 57 (71.3%) patients with positive hepatitis B virus and 23 (28.7%) patients with negative hepatitis B virus. Also, 62 (77.5%) patients had concomitant disease and 18 (22.5%) patients did not have the concomitant disease. Thirty (37.5%) patients received targeted therapy, while 50 (62.5%) patients did not receive targeted therapy. In 56 (70%) patients, the tumor diameter was greater than 5 cm, and in 24 (30%) patients, the diameter was less than 5 cm. Of these, 13 (16.3%) patients had AFP level ≥20 ng/mL, 23 (28.7%) had AFP level of 20–400 ng/mL, and 44 (55%) had AFP level ≥400 ng/mL. Moreover, 32.5% patients (26/80) had low platelet count (<100 × 10^9^/L) and 67.5% (54/80) had normal level (≥100 × 10^9^/L). Further, 36.3% patients (29/80) had low hemoglobin level (<120 g/L) and 63.7% (51/80) had normal level (≥120 g/L). Also, 42.5% cases (34/80) had high total bilirubin level (≥20 μmol/L) and 57.5% (46/80) had a normal level (<20 μmol/L). Moreover, 62.5% patients (50/80) had low albumin level (<35 g/L) and 37.5% (30/80) had normal level (≥35 g/L). Also, 30/80 patients (37.5%) had high ALT level (≥50 U/L) and 50/80 (62.5%) had a normal level (<50 U/L). Further, 46/80 patients (57.5%) had high AST level (≥50 U/L) and 34/80 (42.5%) had a normal level (<50 U/L).

**Table 1:** Patient characteristics

Characteristics		n (%)
Age, y Gender RT BED, Gy Types HBsAg Child Pugh classification PS (ECOG) Concomitant disease Target therapy Center location Diameter, cm AFP, ng/L PLT, 10^9^/L HGB, g/L TBIL, μmol/L Albumin, g/L ALT, U/L AST, U/L	≥54/<54 Male/Female A-RT/2D-RT/Non-RT ≥58.9 /<58.9/0 I /II/III/IV Negative/Positive A/B 0–1/2–3 Yes/No Yes/No Right/Left ≥5/<5 ≤20/20 ~ 400/≥400 ≥100/<100 ≥120/<120 ≥20/<20 ≥35/<35 ≥50/<50 ≥50/<50	41(51.2)/39(48.8) 75(93.7)/5(6.3) 54(67.5)/10(12.5)/16(20.0) 42(52.5)/22(27.5)/16(20.0) 12(15.0)/28(35.0)/18(22.5)/22(27.5) 23(28.7)/57(71.3) 50(62.5)/30(37.5) 59(73.8)/21(26.2) 62(77.5)/18(22.5) 30(37.5)/50(62.5) 60(75.0)/20(25.0) 56(70.0)/24(30.0) 13(16.3)/23(28.7)/44(55.0) 54(67.5)/26(32.5) 51(63.7)/29(36.3) 34(37.5)/46(57.5) 30(37.5)/50(62.5) 30(37.5)/50(62.5) 46(57.5)/34(42.5)

*RT* radiotherapy, *A*-*RT* accurate radiotherapy, *2D*-*RT* two-dimensional radiotherapy, *Non*-*RT* non-radiotherapy, *BED* biologically effective dose, *Types* types of tumor thrombi, *HBsAg* hepatitis B surface antigen, *PS* performance status, *ECOG* Eastern Cooperative Oncology Group; Concomitant disease include hepatitis and hepatocirrhosis; Targeted therapy: sorafenib; *AFP* alpha–fetoprotein, *PLT* platelet, *HGB* Hemoglobin, *TBIL* total bilirubin, *ALT* alanine aminotransferase, *AST* aspartate aminotransferase

### Survival outcomes

**Fig. 1:**
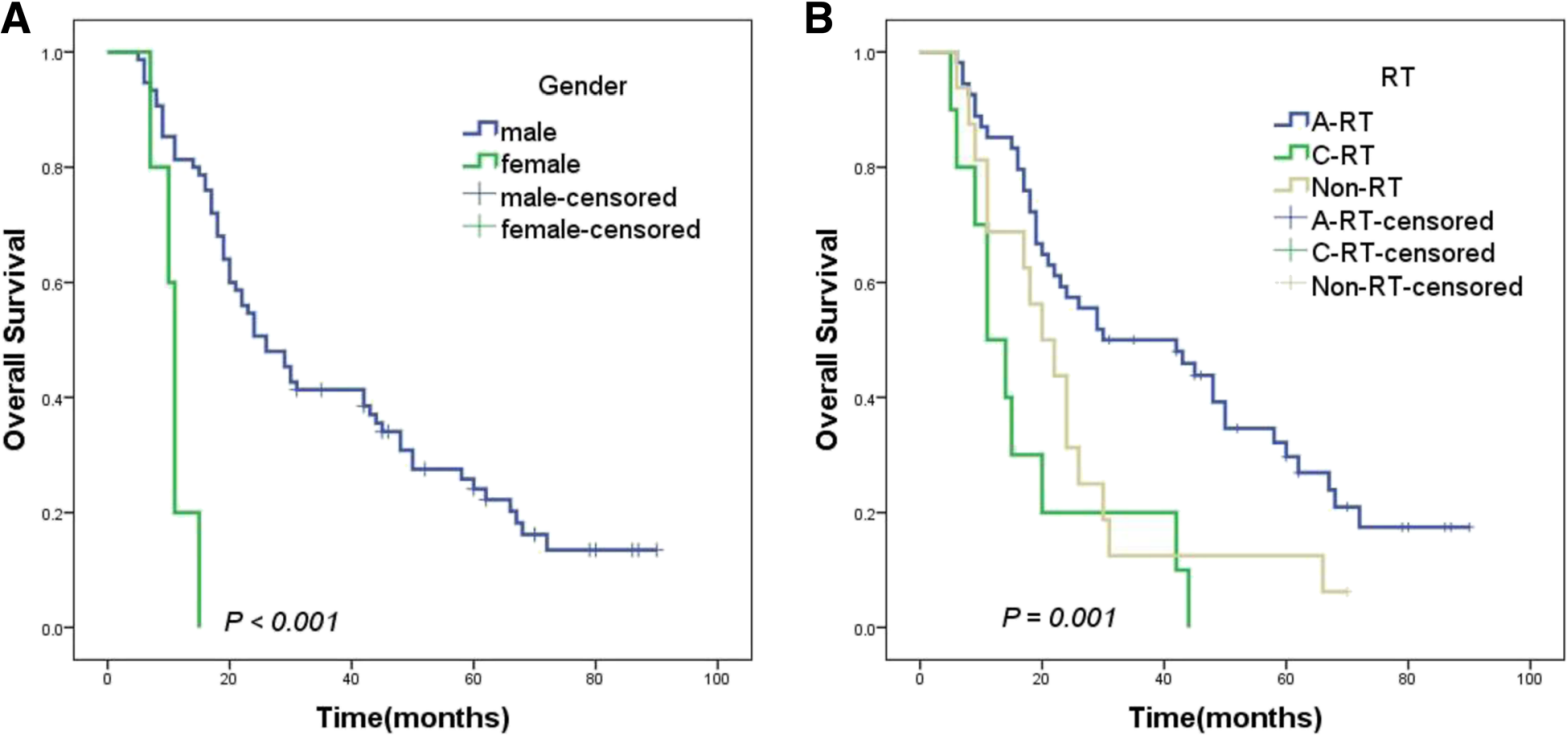
Kaplan–Meier analysis of OS in 80 patients with a tumor thrombus according to (**a**) gender (male: *blue*; female: *green*, *P* < 0.001); (**b**) RT (A-RT: *blue*; 2D-RT: *green*; non-RT: *brown*; *P* = 0.001)

**Fig. 2:**
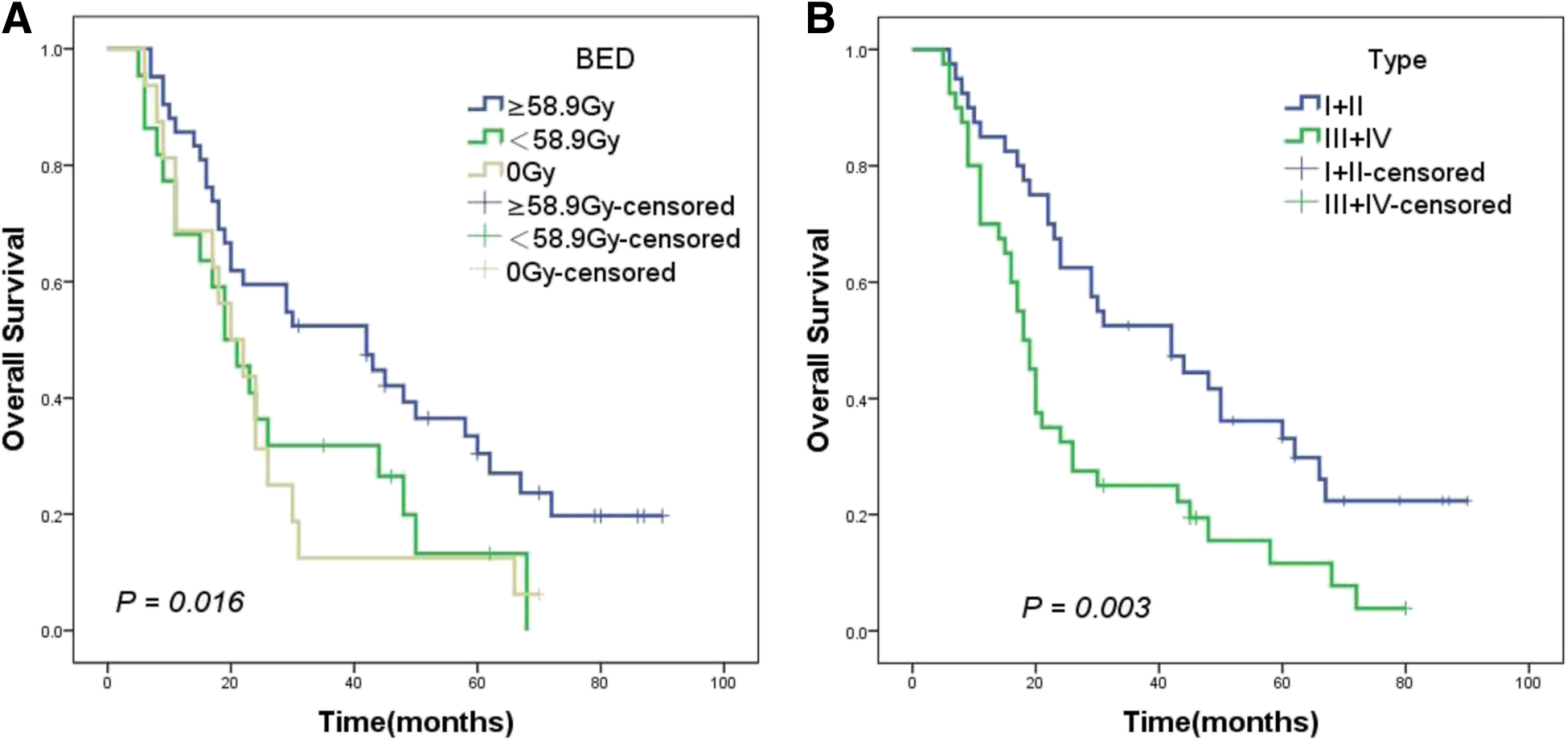
Kaplan–Meier analysis of OS in 80 patients with a tumor thrombus according to (**a**) BED (≥58.9 Gy: *blue*; <58.9 Gy: *green*; *P* = 0.002); (**b**) type (I + II: *blue*; III + IV: *green*; *P* = 0.003)

**Fig. 3:**
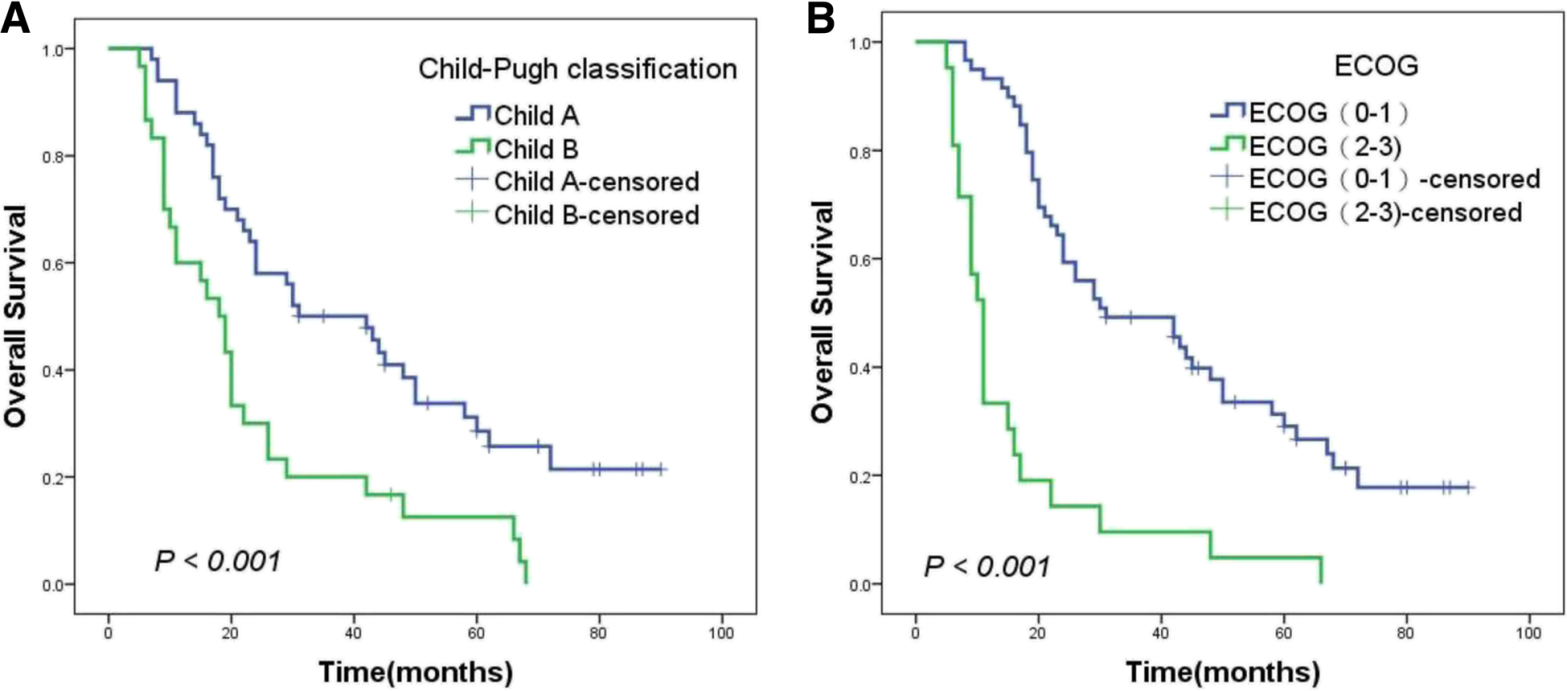
Kaplan–Meier analysis of OS in 80 patients with a tumor thrombus according to (**a**) Child-Pugh classification (Class A: *blue*; Class B: *green*; *P* < 0.001); (**b**) ECOG (0–1: *blue*; 2–3: *green*; *P* < 0.001)

The median OS (mOS) was 24 months (range, 6–90 months). The 1-, 3-, and 5-year OS rates were 77.6%, 37.6%, and 18.8%, respectively. The results of univariate analysis are summarized in Table 2. On univariate analysis, gender, radiotherapy method, BED, types of tumor thrombi, Child-Pugh classification, PS (ECOG) and total bilirubin were considered to be the prognostic factors of survival in patients with a tumor thrombus (Figs. 1, 2 and 3,* P* < 0.05). The result of the multivariate analysis showed that gender, BED, Child-Pugh classification, PS (ECOG) and types of tumor thrombi were independent prognostic factors of OS (Table 3, *P* < 0.05).

**Table 2:** Univariate analysis of overall survival

Variables		n	mOS (month)	χ^2^	*P*
Age, y	≥54/<54	41/39	26/20	1.405	0.236
Gender	Male/Female	75/5	26/11	16.305	<0.001
RT	A-RT/C-RT/Non-RT	54/10/16	30/11/20	14.912	0.001
	A-RT/C-RT	54/10	30/10	13.757	<0.001
	A-RT/Non-RT	54/16	30/20	4.578	0.032
	C-RT/Non-RT	10/16	11/20	1.197	0.274
BED	≥58.9 Gy/<58.9 Gy/0 Gy ≥58.9 Gy/<58.9 Gy ≥58.9 Gy/0 Gy <58.9 Gy/0 Gy	42/22/16 42/22 42/16 22/16	42/19/20 42/19 42/20 19/20	5.763 3.890 4.740 0.048	0.016 0.049 0.029 0.827
Types HBsAg Child Pugh classification	I + II/III + IV Negative/Positive A/B	12 + 28/18 + 22 23/57 50/30	42/31/19/17 30/21 31/18	8.757 0.392 12.682	0.003 0.531 <0.001
PS (ECOG) Concomitant disease	0–1/2–3 Yes/No	59/21 62/18	31/11 23/29	30.943 0.567	<0.001 0.451
Target therapy Center location	Yes/No Right/Left	30/50 60/20	24/23 23/21	0.202 0.223	0.653 0.637
diameter, cm AFP, ng/L	≥5/<5 ≤20/20 ~ 400/≥400 ≤20/20 ~ 400 ≤20/≥400 20 ~ 400/≥400	56/24 13/23/44 13/23 13/44 23/44	21/26 43/30/21 43/30 43/21 30/21	1.001 1.787 1.825 0.015 1.412	0.371 0.409 0.177 0.904 0.235
PLT, 10^9^/L HGB, g/L TBIL, μmol/L Albumin, g/L ALT, U/L AST, U/L	≥100/<100 ≥120/<120 ≥20/<20 ≥35/<35 ≥50/<50 ≥50/<50	54/26 51/29 34/46 30/50 30/50 46/34	26/20 26/21 20/31 22/24 20/26 20/29	0.258 0.019 4.263 0.115 1.777 0.010	0.611 0.891 0.039 0.735 0.182 0.975

*RT* radiotherapy, *A*-*RT* accurate radiotherapy, *2D*-*RT* two-dimensional radiotherapy, *Non-RT* non-radiotherapy, *BED* biologically effective dose, *Types* types of tumor thrombi, *HBsAg* hepatitis B surface antigen, *PS* performance status, *ECOG* Eastern Cooperative Oncology Group; Concomitant disease include hepatitis and hepatocirrhosis; Targeted therapy: sorafenib; *AFP* alpha–fetoprotein, *PLT* platelet, *HGB* Hemoglobin, *TBIL* total bilirubin, *ALT* alanine aminotransferase, *AST* aspartate aminotransferase, *mOS* median overall survival

**Table 3:** Multivariate analysis of overall survival

Variables		n	HR (95% CI)	*P*
Gender	Male/Female	75/5	3.982(1.178–13.462)	0.26
RT	A-RT/2D-RT/Non-RT	54/10/16		0.198
	A-RT/Non-RT 2D-RT/Non-RT	54/16 10/16	0.954(0.187–4.862) 2.135(0.365–12.478)	0.955 0.400
BED, Gy	≥58.9/<58.9/0 Gy	42/22/16		0.039
	≥58.9/0 Gy <58.9/0 Gy	42/16 22/16	0.390(0.075–2.034) 0.971(0.175–5.388)	0.264 0.973
Types Child Pugh classification PS (ECOG) TBIL, μmol/L	I + II/III + IV A/B 0–1/2–3 ≥20/<20	40/40 50/30 59/21 34/46	1.363(1.033–1.798) 1.992(1.115–3.558) 2.820(1.401–5.673) 1.029(0.568–1.866)	0.029 0.020 0.004 0.925

*A-RT* accurate radiotherapy, *2D-RT* two-dimensional radiotherapy, *Non-RT* non-radiotherapy, *BED* biologically effective dose, *Types* types of tumor thrombus, *TBIL* total bilirubin, *CI* confidence interval, *HR* hazard ratio

### Treatment response

The results of radiotherapeutic response are summarized in Table 4. Complete response (CR) was observed in 8 patients (12.5%), partial response (PR) in 28 patients (43.8%), stable disease (SD) in 17 patients (26.6%), progressive disease (PD) in 7 patients (10.9%), and not evaluable in 4 cases (6.2%). The objective RR was 56.3%. For high BED group, CR was achieved in 6 patients (15.4%), PR in 21 patients (53.8%), SD in 9 patients 23.1%), and PD in 3 patients (7.7%). For low BED group, CR was noted in 2 patients (9.6%), PR in 7 patients (33.3%), SD in 8 patients (38.1%), and PD in 4 patients (19.0%). The response rate of the patients in the high BED group (69.2%) was significantly higher than that in the low BED group (42.9%) (*P* = 0.042).

**Table 4:** Radiotherapeutic response

	n	Not evaluated	CR(n)	PR(n)	SD(n)	PD(n)	RR(%)	*P*
High BED group Low BED group All patients	42 22 64	3 1 4	6 2 8	21 7 28	9 8 17	3 4 7	69.2 42.9 56.3	0.042

*BED* biologically effective dose, *CR* complete response, *PR* partial response, *SD* stable disease, *PD* progressive disease, *RR* response rate

### Toxicity

Fifty-one (79.7%) adverse events occurred in 64 patients who received RT, of these, 42 (77.8%) adverse events occurred in the A-RT group and 9 (90.0%) adverse events occurred in the 2D-RT group. There were 5 patients had Grade 3 toxicity but none of patients experienced Grade 4–5 toxicity. Grade 3 marrow suppression occurred in three patients, elevated aminotransferase levels in one patient, and one patient with upper gastrointestinal hemorrhage. In the A-RT group, 24 patients (44.4%) had Grade 1 toxicity, 15 patients (27.8%) with adverse events in Grade 2, and the 3 patients (5.56%) appeared adverse events of Grade 3. In the 2D-RT group, 3 patients (30.0%) had Grade 1 toxicity, 4 patients (40.0%) were confirmed as adverse events in Grade 2, and 2 patients (20.0%) appeared adverse events of Grade 3. No classic radiation-induced liver disease (RILD) was observed in the study.

## Discussion

To date no credible evidence is available for establishing an optimal treatment strategy for HCC with a tumor thrombus. The present study showed that gender, radiotherapy method, BED, and types of tumor thrombi affect OS, and the 1-, 3-, and 5-year OS rates were 77.6%, 37.6%, and 18.8%, respectively. The mOS was reported to be shorter than 3 months for patients with HCC having a tumor thrombus treated with supportive treatment only [15].

A few previous studies did not consider gender as a significant prognostic factor in the univariate or multivariate analysis [18, 19]. A retrospective study of 10,608 patients conducted by Norris Comprehensive Cancer Center demonstrated no gender-related difference in survival in Asian patients with HCC [20]. However, an autopsy study showed that a tumor thrombus was more common in female patients, and hormonal factors might play a role in neoplastic vascular invasion [21]. Further studies found that ovarian hormones could inhibit hepatocarcinogenesis [22]. Menopause presented a growing estrogen deficiency, and this change might contribute to increased susceptibility to HCC [23]. All female patients were diagnosed with HCC after menopause in the present study. Also, gender was regarded as an independent prognostic factor, while males had a longer mOS than that of the females (26 vs 11 months, Fig. 1a, *P* < 0.001). The small number of female patients (6.3%, 5/80) might have led to the statistical bias.

Although sorafenib is recommended in the guideline as the only treatment for patients with HCC having a tumor thrombus [24], several studies have suggested that patients could benefit from RT [4, 5, 11, 15, 25]. Tanaka et al. suggested that RT was an effective treatment without serious adverse events for patients with HCC involving invasion to intrahepatic large vessels [26]. A previous study showed that the mOS was only 13.1 months for 2D-RT alone [27]. Nowadays, TACE had been considered as a first-line therapy for unresectable HCC [28]. Compared with TACE alone, 3D-CRT combined with TACE could evidently improve the outcomes (mOS: 11.0 vs 4.8 months, *P* < 0.001) for patients with HCC having a tumor thrombus [29]. However, all patients in the present study received TACE. The mOS for A-RT, non-RT, and 2D-RT groups were 30, 20, and 11 months, respectively. Also, the 3-year OS rates for A-RT and 2D-RT groups were 45.3% and 20%, respectively (*P* = 0.001, Fig. 1b). In our study, ten patients had received 2D-RT, in which nine patients (9/10) had radiation related toxicity. Of these, 4 patients (40.0%) confirmed as Grade 2 adverse events, and 2 patients (20.0%) appeared Grade 3 adverse events. Grade 3 toxicities included upper gastrointestinal hemorrhage and elevated aminotransferase levels. The poor outcome of patients with 2D-RT might be attributed to the toxicities.

The optimal radiation dose for HCC with a tumor thrombus remains debatable. The radiation dose ranged from 30–72 Gy [30], which aroused people’s attention for exploring the optimal dose of RT for patients with HCC having a tumor thrombus. As the tolerance dose of the entire liver is limited, it is challenging to attain the tumoricidal dose for HCC with a favorable toxicity profile [31]. However, delivering tumoricidal doses to the partial liver has become feasible with the great advances in radiation technology [32]. Chen et al. [33] reported that 3D-CRT could reduce the risk of radiation-induced liver disease in consideration of a lower mean dose (Dmean). Toya et al. [34] found that the response rate in the high-dose (BED ≥ 58 Gy) and low-dose groups (BED < 58 Gy) for a tumor thrombus was 54.6% and 20.0%, respectively (*P* = 0.034). In our study, the response rate of tumor thrombus in the high BED group (69.2%) was significantly higher than that in the low BED group (42.9%) (*P* = 0.042, Table 4). The median BED was 58.9 Gy in the present study. The mOS for the high-BED group was better than that for the low-BED group and non-RT group (42 months vs.19 months and 20months, *P* = 0.016, Fig. 2a).

The prognosis of patients with different types of tumor thrombi was different [35]. Similarly, the median OS in the present study for types I, II, III, and IV tumor thrombi was 42, 31, 19, and 17 months, respectively. Also, the types I and II had a favorable survival outcome compared with the types III and IV (*P* = 0.003, Fig. 2b). Zhang et al. [36] found that the OS for patients with HCC having a tumor thrombus differed significantly with the types of treatment strategies and the extent of tumor thrombus. Unfortunately, limited data focus on selecting an appropriate treatment strategy for a specific type of tumor thrombus. A study of 1580 patients implemented by the Eastern Hepatobiliary Surgery Hospital suggested that surgery was more suitable for types I and II patients with a Child-Pugh A liver function, while the combination of TACE with RT could be more suitable for type III patients [19]. Another study of 158 patients demonstrated a survival advantage with EBRT for patients with type IV; the EBRT group had a higher median OS compared with the non-EBRT group (4 vs. 8 months) [15]. Pretherapy risk stratification of a tumor thrombus and selecting the optimal treatment strategies for patients with a tumor thrombus might be more conducive to prolong survival and might provide an effective way to evaluate patient’s prognosis.

Child-Pugh classification was often used to evaluate liver function of HCC. Some researchers found that Child-Pugh classification is the prognostic factors associated with OS and patients with class A survive longer than class B (mOS: 31 months vs. 12 months, *P* < 0.05) [37]. We also found that the OS of 50 (62.5%) patients with class A was longer than the other patients with class B (mOS: 31 months vs. 18 months, *P* < 0.001). The PS was found to be an important factor affecting the OS of HCC patients with tumor thrombus (*P* < 0.05) [38]. In our analysis, the OS was observed to be significantly different between patients with good PS (ECOG 0–1) and poor PS (ECOG 2–3) (mOS: 31 months vs. 11 months, *P* < 0.001).

This retrospective single-institution study had some limitations. First, the sample size of the study was small. Second, the gender proportion should have been noted to avoid any statistical bias.

## Conclusion

In conclusion, the present study estimated the prognostic factors influencing OS in HCC with a tumor thrombus. Although the prognosis for HCC with a tumor thrombus remained poor, high-BED palliative radiotherapy for a tumor thrombus might improve the long-term outcomes. Prospective studies to determine an appropriate BED for HCC with a thrombus are warranted in the future.

## Abbreviations

2D-RT: Two-dimensional radiotherapy
3D-CRT: Three-dimensional conformal radiation therapy
AFP: Alpha-fetoprotein
A-RT: Accurate radiotherapy
BED: Biologically effective dose
CT: Computed tomography
CTCAE: Terminology Criteria for Adverse Events
CTV: Clinical target volume
Dmean: Mean dose
EBRT: External-beam radiation therapy
ECOG: Eastern Cooperative Oncology Group
GTV: Gross tumor volume
HCC: Hepatocellular carcinoma
mOS: Median overall survival
mRECIST: Modified Response Evaluation Criteria in Solid Tumors
MRI: Magnetic resonance imaging
OS: Overall survival
PS: Performance status
PTV: Planning target volume
RILD: Radiation-induced liver disease
RT: Radiotherapy
TACE: Transcatheter arterial chemoembolization
